# Hangeshashinto Inhibits *Porphyromonas gingivalis* Pathogen-Associated Molecular Patterns-Mediated IL-6 and IL-8 Production through Toll-Like Receptors in CAL27 Cells

**DOI:** 10.1155/2024/9866670

**Published:** 2024-04-18

**Authors:** Hourei Oh, Yoshimasa Makita, Kazuya Masuno, Yasuhiro Imamura

**Affiliations:** ^1^Center of Innovation in Dental Education, Osaka Dental University, Osaka 573-1121, Japan; ^2^Department of Chemistry, Osaka Dental University, Osaka 573-1121, Japan; ^3^Department of Pharmacology, Matsumoto Dental University, Nagano 399-0781, Japan

## Abstract

While previous reports have established the anti-inflammatory effects of hangeshashinto, the intracellular signal transduction pathways involved have yet to be elucidated. We aim to employ an experimental system using oral cancer cells to assess the impact of hangeshashinto on intracellular signal transduction pathways in response to stimulation by *Porphyromonas gingivalis* pathogen-associated molecular patterns (PAMP). Hangeshashinto demonstrated the ability to inhibit the production of interleukin (IL)-6 and IL-8 induced by *P. gingivalis* PAMP. Furthermore, hangeshashinto suppressed the activation of the IL-6 promoter stimulated by PAMP. Hangeshashinto, like Toll-like receptor (TLR) signaling inhibitors (resatorvid and C29) and an immunosuppressant (dexamethasone), exhibited the ability to suppress TLR-mediated activation of the transcription factor nuclear factor-*κ*B (NF-*κ*B) in response to PAMP stimulation. This study suggests that the anti-inflammatory effects of hangeshashinto may be attributed to the inhibition of TLR signal transduction pathways including NF-*κ*B activation, thereby suppressing NF-*κ*B-dependent gene expression.

## 1. Introduction

Kampo is a traditional Japanese medicine with its roots in Chinese medicine. It encompasses herbal remedies and is currently prescribed by physicians and dentists in Japan [1]. Among the various kampo medicines, hangeshashinto holds a significant place and can be traced back to ancient Chinese medical texts such as “Shang Han Lun” and “Jin Gui Yao Lue,” compiled in the early 3rd century [2]. Hangeshashinto consists of seven herbal extracts, including pinellia tuber, coptis rhizome, scutellaria root, processed ginger, glycyrrhiza, ginseng, and jujube. It has gained approval as a prescribed medicine in both Japan and China. Notably, hangeshashinto has been increasingly prescribed in Japan for the treatment of oral mucositis, and clinical studies employing randomized place-bo-controlled double-blind trials have demonstrated its efficacy in managing oral mucositis caused by chemotherapy for colon and stomach cancers [3]. In clinical practice, hangeshashinto is administered three times a day, with a dosage of 4.5 grams each time for oral mucositis treatment. Our research group has been at the forefront of investigating the anti-inflammatory effects of hangeshashinto. Specifically, we have observed its ability to inhibit lipopolysaccharide (LPS)-induced prostaglandin E2 (PGE2) production, reduce the secretion of interleukin (IL)-6 and IL-8, suppress the activity of cyclooxygenase (COX)-1 and -2, and decrease the expression of cytosolic phospholipase (PL) A2 and LPS-induced COX-2 in human gingival fibroblasts [4]. Subsequent in vitro [5, 6] and in vivo experiments [7–9] have further shed light on the underlying mechanisms by which hangeshashinto improves oral mucositis, highlighting its anti-inflammatory and antibacterial properties. While previous reports have established the anti-inflammatory effects of hangeshashinto, the intracellular signal transduction pathways involved have yet to be elucidated. We aim to employ an experimental system using oral cancer cells to assess the impact of hangeshashinto on intracellular signal transduction pathways in response to stimulation by *Porphyromonas gingivalis* (*P. gingivalis*) pathogen-associated molecular patterns (PAMP). *P. gingivalis* is a bacterium associated with periodontitis and serves as a reliable stimulus for IL-6 production in oral cancer cells [10]. An experimental system has been established that utilizes *P. gingivalis* LPS as a model for inflammation, inducing the production of inflammatory cytokines in cultured cells [11]. It is recognized that these inflammatory cytokines exert influence on the development of stomatitis [12]. Our study aims to provide valuable insights into the effects of hangeshashinto on intracellular signaling in response to *P. gingivalis* PAMP stimulation, using an experimental model involving oral cancer cells. This investigation will contribute to a deeper understanding of the mechanisms underlying the therapeutic potential of hangeshashinto in managing oral mucositis and related inflammatory conditions.

## 2. Materials and Methods

### 2.1. Cell Culture

CAL27 cells (oral squamous cell carcinoma, American Type Culture Collection, Virginia, USA) were cultured at 37°C and 5% CO_2_ in Dulbecco's Modified Eagle Medium (DMEM) (manufactured by Nissui Pharmaceutical, Tokyo) containing 10% fetal bovine serum (FBS), 100 units/ml penicillin G, and 100 *μ*g/ml streptomycin.

### 2.2. Reagents

**Figure 1:**
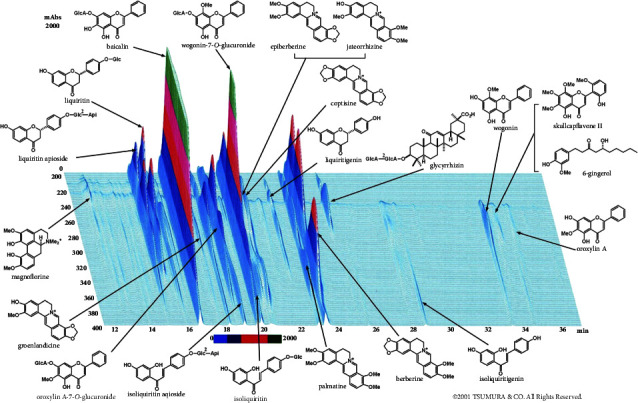
Three-dimensional high-performance liquid chromatography profile of hangeshashinto (provided by Tsumura, Tokyo, Japan).

Hangeshashinto was provided by Tsumura (Tokyo, Japan). *P. gingivalis* LPS (InvivoGen, California, USA, referred to as “PAMP” in this study) (see Imamura et al. [10]). 3-(4,5-dimethylthiazol-2-yl)-2,5-diphenyltetrazolium bromide (MTT) and dexamethasone (Sigma-Aldrich, Missouri, USA); resatorvid and C29 (Selleck Biotech, Kanagawa, Japan); anti-IL-6 antibody and biotinylated anti-IL-6 antibody (Thermo Fisher Scientific (eBioscience), Massachusetts, USA); anti-IL-8 antibody and biotinylated anti-IL-8 antibody (R&D Systems, Minnesota, USA). Chemical composition analysis of hangeshashinto was conducted by Tsumura using three-dimensional high-speed liquid chromatography profiling (Figure 1).

### 2.3. MTT Assay

CAL27 cells (1 × 10^4^) were cultured for 24 hours in concentrations of 10, 100, and 1000 *μ*g/mL of hangeshashinto. Subsequently, the cells were cultured with 0.5 mg/mL MTT for 4 hours. Then, 0.02 N HCl-isopropanol was added to the medium to dissolve and solubilize the formazan product completely. Samples were measured using a Microplate Reader model 550 (Bio-Rad Labs, Inc., Hercules, California, USA) with dual wavelengths of 595/655 nm (test/reference), as previously described [10].

### 2.4. Enzyme-Linked Immunosorbent Assays (ELISAs)

CAL27 cells (1 × 10^4^) were cultured for 24 hours in a mixture of *P. gingivalis* PAMP (100 ng/mL) and hangeshashinto (1000 *μ*g/mL). The levels of IL-6 in the culture medium were measured using anti-IL-6 (1 *μ*g/mL) and biotinylated anti-IL-6 (0.6 *μ*g/mL) antibodies. The levels of IL-8 in the culture medium were also measured using anti-IL-8 (2.5 *μ*g/mL) and biotinylated anti-IL-8 (0.2 *μ*g/mL) antibodies. Samples were measured using a Microplate Reader model 550 with dual wavelengths of 450/655 nm (test/reference). The ELISA procedure was carried out as described in the CytoSet Kit User Manual (Thermo Fisher Scientific (BioSource)) [10].

### 2.5. Transfection and Luciferase Assay

**Figure 2:**
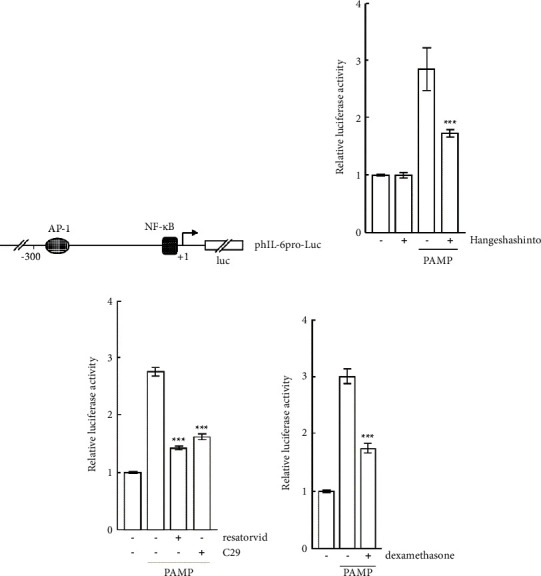
(a) Schematic representation of a reporter plasmid phIL-6pro-Luc containing the IL-6 promoter linked to the luciferase gene (luc). Putative binding sites of transcriptional factors are indicated. Plasmids of phIL-6pro-Luc and pRSV-*β*-gal (standard) were cotransfected into CAL27 cells. Twenty-four hours after transfection, the cells were cultured with hangeshashinto (b), resatorvid and C29 (c), and dexamethasone (d) for 24 hours. Then, the cells were stimulated with PAMP for 6 hours. The cells were lysed, and luciferase and *β*-galactosidase assays were performed. The transcriptional activities indicated fold values of those with no stimulators. Bars represent the means and range of triplicate samples. ^*∗∗∗*^, *p* < 0.001 versus stimulation with PAMP alone (one-way ANOVA with Tukey's test).

**Figure 3:**
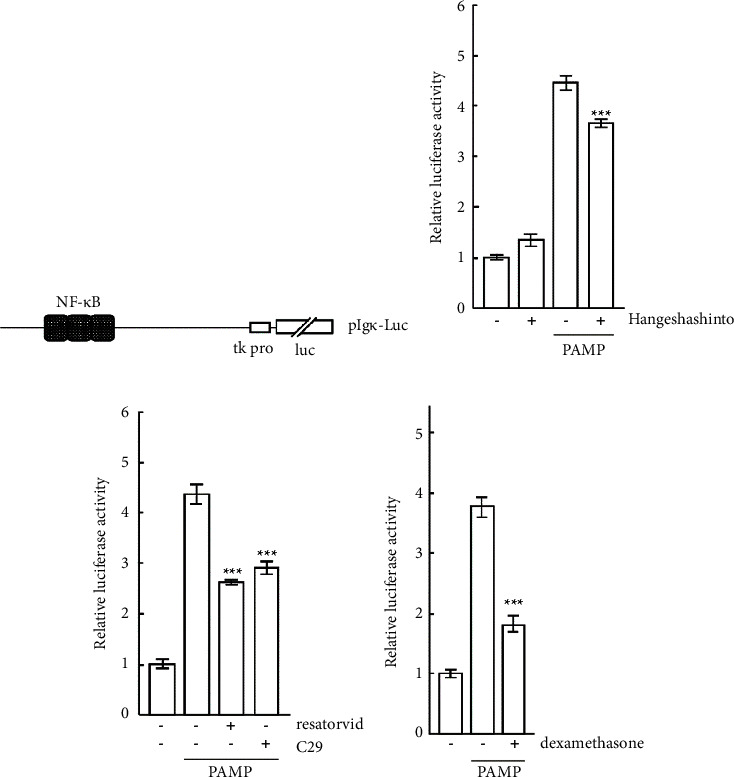
(a) Schematic representation of a reporter plasmid pIg*κ*-Luc containing three copies of an NF-*κ*B binding site and the thymidine kinase promoter (tk pro) linked to the luciferase gene (luc). Plasmids of pIg*κ*-Luc and pRSV-*β*-gal were co-transfected into CAL27 cells. Twenty-four hours after transfection, the cells were cultured with hangeshashinto (b), resatorvid and C29 (c), and dexamethasone (d) for 24 hours. Then, the cells were stimulated with PAMP for 6 hours. The next procedures were performed as described in “materials and methods.” ^*∗∗∗*^, *p* < 0.001 versus stimulation with PAMP alone (one-way ANOVA with Tukey's test).

One microgram of phIL-6pro-Luc (a fusion of the human IL-6 promoter region spanning from position 2636 to 5035 of GenBank Accession Number NG_011640 linked to the luciferase gene (Figure 2(a)) [10], along with 0.1 *μ*g of pRSV-*β*-gal (standard plasmid), was mixed with TransIT-LT1 reagent (Mirus Bio, Wisconsin, USA). Additionally, 1 *μ*g of pIg*κ*-Luc (Figure 3(a)) [10] and 0.1 *μ*g of pRSV-*β*-gal were also mixed with TransIT-LT1 reagent. The mixtures were then added to the cells (3 × 10^5^), which were subsequently cultured for 24 hours. Afterward, the cells were stimulated with hangeshashinto (1000 *μ*g/mL) for 24 hours, followed by stimulation with *P. gingivalis* PAMP (100 ng/mL) for 6 hours. The cells were harvested and lysed. The luciferase and *β*-galactosidase activities in the lysates were measured as previously described [10].

### 2.6. Statistical Analysis

Quantitative data were analyzed using one-way analysis of variance (ANOVA). Data were then statistically analyzed using the Tukey's test (StatMate software (ATMS, Chiba, Japan)). Significant differences were considered statistically significant at *p* < 0.05.

## 3. Results

### 3.1. Effect of Hangeshashinto on Viability of CAL27 Cells

**Figure 4:**
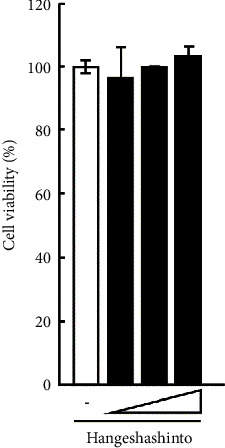
Effect of hangeshashinto on survival of CAL27 cells. CAL27 cells (1 × 10^4^) were cultured with hangeshashinto (10, 100, and 1000 *μ*g/mL) for 24 hours and an MTT assay was performed. Bars represent the means and range of triplicate samples.

We examined the impact of hangeshashinto on the viability of CAL27 cells. CAL27 cells were subjected to a 24-hour culture with hangeshashinto, followed by an MTT assay. As depicted in Figure 4, the presence of hangeshashinto at concentrations up to 1000 *μ*g/mL did not exert any discernible influence on the viability of CAL27 cells. Therefore, the experiments described below were carried out with a concentration of 1000 *μ*g/mL hangeshashinto.

### 3.2. Effects of Hangeshashinto on Proinflammatory Cytokine Production by PAMP Stimulation

**Figure 5:**
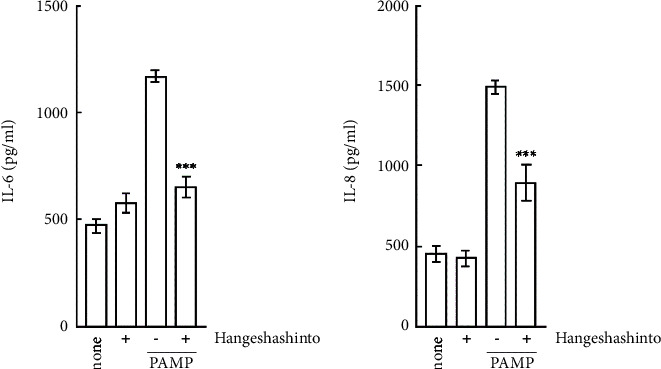
Effects of hangeshashinto on the production of proinflammatory cytokines in CAL27 cells by PAMP. Hangeshashinto (1000 *μ*g/ml) and PAMP (100 ng/ml) were added to CAL27 cells and the cells were cultured for 24 hours. The levels of IL-6 (a) and IL-8 (b) in the culture media were measured by ELISAs. ^*∗∗∗*^, *p* < 0.001 versus stimulation with PAMP alone (one-way ANOVA with Tukey's test).

CAL27 cells were cultured with PAMP stimulation in the presence of hangeshashinto. The amounts of IL-6 and IL-8 in the culture medium were measured using the ELISA method. The results indicate that PAMP stimulation significantly enhanced the production of IL-6 (Figure 5(a), *p* < 0.001) and IL-8 (Figure 5(b), *p* < 0.001) in CAL27 cells. The levels of production approximately 2.5 times for IL-6 and approximately 3 times for IL-8 higher compared to that of unstimulated cells. Interestingly, the presence of hangeshashinto resulted in an approximate 60% reduction in IL-6 production induced by PAMP stimulation. These results suggest that hangeshashinto suppresses the production of proinflammatory cytokines by *P. gingivalis* PAMP in CAL27 cells.

### 3.3. Transcriptional Regulation of the IL-6 Promoter by PAMP in the Presence of Hangeshashinto

The effect of hangeshashinto on the transcriptional activity of the IL-6 promoter was investigated. A reporter plasmid containing the IL-6 promoter linked to the luciferase gene (Figure 2(a)) was transfected into CAL27 cells. The cells were stimulated with PAMP in the presence or absence of hangeshashinto, and a luciferase assay was performed. As shown in Figure 2(b), PAMP enhanced the transcriptional activity of the IL-6 promoter, but hangeshashinto alone did not enhance it. When CAL27 cells were stimulated with PAMP in the presence of hangeshashinto, the level of transcriptional activity significantly decreased by approximately 50% compared to that of PAMP stimulation alone (*p* < 0.001). Resatorvid and C29, Toll-like receptor (TLR) 4 and TLR2 signaling inhibitors, respectively, also significantly repressed the transcriptional activity of the IL-6 promoter upon PAMP stimulation (Figure 2(c), *p* < 0.001). In addition, dexamethasone, which is an immunosuppressant and an anti-inflammatory agent, repressed that activity (Figure 2(d), *p* < 0.001). These results suggest that hangeshashinto inhibits the transcriptional activation of the IL-6 promoter via the TLR signaling pathways.

### 3.4. Nuclear Factor-*κ*B (NF-*κ*B) Activation by PAMP with or without Hangeshashinto

Next, the effect of hangeshashinto on the activation of NF-*κ*B by PAMP stimulation was investigated. An NF-*κ*B-dependent reporter plasmid (Figure 3(a)) was transfected into CAL27 cells. The cells were stimulated with PAMP in the presence or absence of hangeshashinto, and luciferase assays were performed. As shown in Figure 3(b), the level of NF-*κ*B-dependent transcriptional activation by PAMP stimulation was approximately 4.5 times higher than that without PAMP stimulation. When CAL27 cells were stimulated with PAMP in the presence of hangeshashinto, the level of transcriptional activation significantly decreased compared to that in the absence of hangeshashinto (*p* < 0.001). The transcriptional activation with PAMP in the presence of resatorvid or C29 was also significantly suppressed compared to that with PAMP alone (Figure 3(c), *p* < 0.001). Moreover, dexamethasone significantly suppressed the transcriptional activation by stimulating with PAMP in CAL27 cells (Figure 3(d), *p* < 0.001). These results suggest that hangeshashinto inhibits NF-*κ*B activation through the TLR signaling pathways.

## 4. Discussion

Hangeshashinto which has an anti-inflammatory effect is indicated for stomatitis [5, 6]. Recent review studies have demonstrated the high effectiveness of hangeshashinto in the treatment of stomatitis. Currently, hangeshashinto is widely used for preventing and treating oral mucositis [13], as it has anti-inflammatory properties. However, the anti-inflammatory mechanism of action of hangeshashinto is not known in detail. TLR signaling induces the activation of some transcription factors and thereby expresses various genes. Upon binding of ligands (PAMP) to TLRs, in the myeloid differentiation factor 88 (MyD88)-dependent pathway, adaptor proteins such as MyD88 and Toll/IL-1R domain–containing adaptor inducing IFN-*β* (TRIF) are recruited to TLRs [14], resulting in forming myddosome, which contains MyD88 and interleukin 1 receptor associated kinase (IRAK) family [15]. IRAK1 activated by autophosphorylated IRAK4 activates TNF receptor–associated factor 6 (TRAF6) [16]. TRAF6 signaling induces the activation of mitogen-activated protein kinase MAPK (c-Jun N-terminal kinase (JNK) and p38) and NF-*κ*B [17]. Subsequently, activator protein-1 (AP-1, dimers of FOS and JUN) and NF-*κ*B stimulate the expression of proinflammatory cytokines and chemokines [18]. The present finding showed that the NF-*κ*B activation through TLRs by PAMP was observed, which was significantly suppressed by hangeshashinto (Figure 3(b)). A recent study has demonstrated that orento, a traditional Kampo medicine, can inhibit TLR signaling by blocking the interaction between MyD88 and IRAK4, resulting in the suppression of NF-*κ*B activation [10]. It is tempting to speculate that hangeshashinto might also suppress NF-*κ*B activation by affecting somewhere in the TLR signaling pathway. This affects the gene expression of proinflammatory cytokines, such as IL-6. Indeed, the expression of the IL-6 gene is regulated by NF-*κ*B [19] (because there is the NF-*κ*B binding site in the IL-6 promoter (Figure 2(a)), which is suppressed by glucocorticoids, like dexamethasone [20] (Figures 2(d) and 3(d)). The detailed mechanisms of action of hangeshashinto need to be clarified. It has been reported that hangeshashinto inhibits PGE2 production by IL-1*β*-stimulated human oral keratinocytes [9]. This seems to imply that hangeshashinto suppresses NF-*κ*B activation, suppressing the gene expression of COX-2, the rate-limiting enzyme for PGE2 production in the arachidonic acid cascade. In fact, the COX-2 gene is expressed by the NF-*κ*B regulation [21]. Our findings also showed that hangeshashinto represses transcriptional activation of the IL-6 promoter (containing the NF-*κ*B binding site) by PAMP stimulation in CAL27 cells (Figure 2(b)), thus reducing IL-6 production (Figure 5(a)). It is conceivable that hangeshashinto may suppress TLR signaling-mediated NF-*κ*B activation (Figure 3(b), see discussion above). It is well known that the expression of various genes is dependent on (regulated by) NF-*κ*B. In fact, the IL-6 gene expression is increased by NF-*κ*B activation in Vibrio vulnificus infection in human peripheral blood mononuclear cells [22]. Hence, hangeshashinto may be the traditional Kampo medicine for (inflammatory) diseases caused by IL-6 that are expressed in the activation of NF-*κ*B through TLRs. Hangeshashinto is a traditional Japanese herbal formula that contains seven medicinal herbs, including pinellia tuber, coptis rhizome, scutellaria root, processed ginger, glycyrrhiza, ginseng, and jujube [4]. In an in vitro experimental system using human oral keratinocytes (HOK), it has been reported that the expression of the COX-2 gene is suppressed by baicalin and berberine, which are constituents of scutellaria root. Additionally, it is suggested that wogonin, another component of scutellaria root, inhibits the MAPK pathway and suppresses the transcription factor activation factor NF-*κ*B [9]. Animal studies have suggested that constituents of hangeshashinto, such as baicalin from scutellaria root and berberine from coptis rhizome, may have anti-inflammatory effects by suppressing the production of pro-inflammatory cytokines [23]. Moreover, hangeshashinto has been reported to ameliorate chemotherapy-induced oral mucositis by reducing oxidative stress and inhibiting the expression of inflammatory mediators [23]. Further research is needed to elucidate the molecular mechanisms underlying the pharmacological effects of hangeshashinto. In recent years, we conducted a randomized double-blind placebo-controlled trial of hangeshashinto in 16 patients with head and neck cancer who developed oral mucositis due to chemical radiation therapy. The trial demonstrated that gargling with hangeshashinto shortened the healing time of oral mucositis and reduced the severity of mucositis grade [24]. In addition, a study involving 22 terminally ill cancer patients reported that hangeshashinto improved oral dryness and may prevent the development of oral mucositis [25]. Notably, in addition to its anti-inflammatory effects, hangeshashinto has been reported to selectively inhibit the growth of Gram-negative bacteria, demonstrating potential antibacterial properties [26]. The importance of prevention has been recognized in traditional Chinese medicine for over 2000 years, as evidenced by the statement “a wise man treats the disease before it occurs” in The Yellow Emperor's Classic of Medicine. In Oriental medicine, the term “pre-disease” refers to a state of mild symptoms that do not progress to full-blown disease [27]. Therefore, the anti-inflammatory effects of kampo medicine may be viewed as a means of preventing pre-disease from progressing to full-blown disease.

## 5. Conclusions

In conclusion, this study suggests that the anti-inflammatory effects of hangeshashinto may be attributed to the inhibition of TLR signal transduction pathways including NF-*κ*B activation, thereby suppressing NF-*κ*B-dependent gene expression.

## Data Availability

The data used to support the findings of this study are available from the corresponding author upon request.

## Abbreviations

*P. gingivalis*: *Porphyromonas gingivalis*
PAMP: Pathogen-associated molecular patterns
NF-*κ*B: Nuclear factor-*κ*B
TLR: Toll-like receptor
LPS: Lipopolysaccharide
PGE2: Prostaglandin E2
IL: Interleukin
COX: Cyclooxygenase
PL: Phospholipase
DMEM: Dulbecco's modified eagle medium
FBS: Fetal bovine serum
MTT: 3-(4,5-dimethylthiazol-2-yl)-2,5-diphenyltetrazolium bromide
ANOVA: Analysis of variance
MyD88: Myeloid differentiation factor 88
TRIF: Toll/IL-1R domain–containing adaptor inducing IFN-*β*
IRAK: Interleukin 1 receptor-associated kinase
TRAF6: TNF receptor-associated factor 6
MAPK: Mitogen-activated protein kinase
JNK: c-Jun N-terminal kinase
AP-1: Activator protein-1.

## References

[1] Wang P. L., Kaneko A. (2018). Introduction to Kampo medicine for dental treatment—oral pharmacotherapy that utilizes the advantages of Western and Kampo medicines. *Japanese Dental Science Review*.

[2] Sakai Y., Namiki T. (2012). Kampo formulae classifications in kenzo Okuda^|^apos;s kampo koho yoho kaisetsu. *Kampo Medicine*.

[3] Matsuda C., Munemoto Y., Mishima H. (2015). Double-blind, placebo-controlled, randomized phase II study of TJ-14 (Hangeshashinto) for infusional fluorinated-pyrimidine-based colorectal cancer chemotherapy-induced oral mucositis. *Cancer Chemotherapy and Pharmacology*.

[4] Nakazono Y., Ara T., Fujinami Y., Hattori T., Wang P. L. (2010). Preventive effects of a Kampo medicine, hangeshashinto on inflammatory responses in lipopolysaccharide-treated human gingival fibroblasts. *Journal of Hard Tissue Biology*.

[5] Ozawa N., Onda T., Hayashi K., Honda H., Shibahara T. (2020). Effects of topical hangeshashinto (TJ-14) on chemotherapy-induced oral mucositis. *Cancer Management and Research*.

[6] Ohnishi S., Takeda H. (2015). Herbal medicines for the treatment of cancer chemotherapy-induced side effects. *Frontiers in Pharmacology*.

[7] Miyano K., Eto M., Hitomi S. (2020). The Japanese herbal medicine hangeshashinto enhances oral keratinocyte migration to facilitate healing of chemotherapy-induced oral ulcerative mucositis. *Scientific Reports*.

[8] Hitomi S. S., Ono K., Terawaki K. (2017). [6]-Gingerol and [6]-shogaol, active ingredients of the traditional Japanese medicine hangeshashinto, relief oral ulcerative mucositis-induced pain via action on Na^+^ channels. *Pharmacological Research*.

[9] Kono T., Kaneko A., Matsumoto C. (2014). Multitargeted effects of hangeshashinto for treatment of chemotherapy-induced oral mucositis on inducible prostaglandin E2 production in human oral keratinocytes. *Integrative Cancer Therapies*.

[10] Imamura Y., Makita Y., Masuno K., Oh H. (2022). Inhibitory mechanism of IL-6 production by orento in oral squamous cell carcinoma cell line CAL27 stimulated by pathogen-associated molecular patterns from periodontopathogenic *Porphyromonas gingivalis*. *International Journal of Molecular Sciences*.

[11] Wang P. L., Ohura K. (2002). *Porphyromonas gingivalis* lipopolysaccharide signaling in gingival fibroblasts–CD14 and toll-like receptors. *Critical Reviews in Oral Biology & Medicine*.

[12] Sonis S. T. (2009). Mucositis: the impact, biology and therapeutic opportunities of oral mucositis. *Oral Oncology*.

[13] Wang Y. T., Ren Y., Xiao C., Liu H., Fu X., You F.-M. (2021). Hangeshashinto for preventing oral mucositis in patients receiving cancer treatment: protocol for a systematic review and meta-analysis. *BMJ Open*.

[14] Snyder M. L., Snyder G. A. (2020). Cobbling together the myddosome. *Structure*.

[15] Deliz-Aguirre R., Cao F., Gerpott F. H. U. (2021). MyD88 oligomer size functions as a physical threshold to trigger IL1R myddosome signaling. *The Journal of Cell Biology*.

[16] Ferrao R., Zhou H., Shan Y. (2014). IRAK4 dimerization and trans-autophosphorylation are induced by myddosome assembly. *Molecular Cell*.

[17] Shang L., Deng D., Buskermolen J. K. (2019). Commensal and pathogenic biofilms alter toll-like receptor signaling in reconstructed human gingiva. *Frontiers in Cellular and Infection Microbiology*.

[18] Beck I. M. E., Vanden Berghe W., Vermeulen L., Yamamoto K. R., Haegeman G., De Bosscher K. (2009). Crosstalk in inflammation: the interplay of glucocorticoid receptor-based mechanisms and kinases and phosphatases. *Endocrine Reviews*.

[19] Libermann T. A., Baltimore D. (1990). Activation of interleukin-6 gene expression through the NF-kappa B transcription factor. *Molecular and Cellular Biology*.

[20] De Bosscher K., Vanden Berghe W., Vermeulen L., Plaisance S., Boone E., Haegeman G. (2000). Glucocorticoids repress NF-*κ*B-driven genes by disturbing the interaction of p65 with the basal transcription machinery, irrespective of coactivator levels in the cell. *Proceedings of the National Academy of Sciences*.

[21] Lim J. W., Kim H., Kim K. H. (2001). Nuclear factor-kappaB regulates cyclooxygenase-2 expression and cell proliferation in human gastric cancer cells. *Laboratory Investigation*.

[22] Chuang C. C., Chuang Y. C., Chang W. T. (2010). Macrophage migration inhibitory factor regulates interleukin-6 production by facilitating nuclear factor-kappa B activation during *Vibrio vulnificus* infection. *BMC Immunology*.

[23] Hitomi S., Ono K., Yamaguchi K. (2016). The traditional Japanese medicine hangeshashinto alleviates oral ulcer-induced pain in a rat model. *Archives of Oral Biology*.

[24] Taira K., Fujiwara K., Fukuhara T., Koyama S., Takeuchi H. (2020). The effect of hangeshashinto on oral mucositis caused by induction chemotherapy in patients with head and neck cancer. *Yonago Acta Medica*.

[25] Murakami S., Igarashi A., Miyano K. (2019). Effects of oral rinse with hangeshashinto alone and hangeshashinto with honey for oral discomfort in terminally-ill cancer patients. *Palliative Care Research*.

[26] Fukamachi H., Matsumoto C., Omiya Y. (2015). Effects of hangeshashinto on growth of oral microorganisms. *Evidence-based Complementary and Alternative Medicine*.

[27] Ni M. (1995). *The Yellow Emperor’s Classic of Medicine: A New Translation of the Neijing Suwen with Commentary*.

